# Systemic TNF-α produces acute cognitive dysfunction and exaggerated sickness behavior when superimposed upon progressive neurodegeneration

**DOI:** 10.1016/j.bbi.2016.09.011

**Published:** 2017-01

**Authors:** Edel Hennessy, Shane Gormley, Ana Belen Lopez-Rodriguez, Caoimhe Murray, Carol Murray, Colm Cunningham

**Affiliations:** School of Biochemistry & Immunology, Trinity College Institute of Neuroscience, Trinity College Dublin, Dublin 2, Ireland

**Keywords:** TNF-α, Systemic inflammation, Microglia, Sickness, Behavior, Dementia, Delirium

## Abstract

Inflammation influences chronic neurodegeneration but its precise roles are not yet clear. Systemic inflammation caused by infection, trauma or co-morbidity can alter the brain’s inflammatory status, produce acute cognitive impairments, such as delirium, and drive new pathology and accelerated decline. Consistent with this, elevated systemic TNF-α is associated with more rapid cognitive decline over 6 months in Alzheimer’s disease patients. In the current study we challenged normal animals and those with existing progressive neurodegeneration (ME7 prion disease) with TNF-α (i.p.) to test the hypothesis that this cytokine has differential effects on cognitive function, sickness behavior and features of underlying pathology contingent on the animals’ baseline condition. TNF-α (50 μg/kg) had no impact on performance of normal animals (normal brain homogenate; NBH) on working memory (T-maze) but produced acute impairments in ME7 animals similarly challenged. Plasma TNF-α and CCL2 levels were equivalent in NBH and ME7 TNF-challenged animals but hippocampal and hypothalamic transcription of IL-1β, TNF-α and CCL2 and translation of IL-1β were higher in ME7 + TNF-α than NBH + TNF-α animals. TNF-α produced an exaggerated sickness behavior response (hypothermia, weight loss, inactivity) in ME7 animals compared to that in NBH animals. However a single challenge with this dose was not sufficient to produce *de novo* neuronal death, synaptic loss or tau hyperphosphorylation that was distinguishable from that arising from ME7 alone. The data indicate that acutely elevated TNF-α has robust acute effects on brain function, selectively in the degenerating brain, but more sustained levels may be required to significantly impact on underlying neurodegeneration.

## Highlights

Mice with prior hippocampal neurodegeneration are more susceptible to systemic TNF-α, displaying:

• Acute working memory deficits not present in normal animals similarly challenged.
• Exaggerated sickness behavior with respect to normal TNF-challenged animals.
• Heightened brain expression of TNF-α mRNA and IL-1β mRNA and protein.

## Graphical abstract

**Figure:**
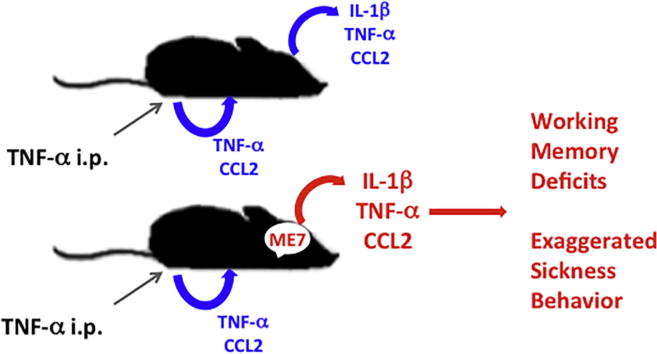

## 1 Introduction

It has become clear that inflammation contributes to chronic neurodegeneration but its precise roles are not yet clear and there remains no effective treatment for slowing the progression of chronic conditions such as Alzheimer’s and Parkinson’s disease. There is substantial epidemiological evidence that inflammatory co-morbidities are significant risk factors for dementia (Yaffe et al., 2004, Dunn et al., 2005, Reitz et al., 2011) and taking non-steroidal anti-inflammatory drugs protects against subsequent development of AD (Etminan et al., 2003). Consistent with a role of co-morbid inflammation, we and others have shown that systemic inflammation can robustly alter brain inflammatory status, inducing a switching of microglial phenotype from ‘primed’ to activated or from M2 to M1, with the consequence of acutely elevated brain levels of IL-1β (Cunningham et al., 2005, Godbout et al., 2005, Pott-Godoy et al., 2008). The pro-inflammatory cytokine IL-1β has been shown to contribute to impaired cognitive function and decreased neuronal viability (Relton and Rothwell, 1992, Vereker et al., 2001, Balosso et al., 2008, Pott-Godoy et al., 2008, Vezzani et al., 2011) in a number of model systems. In animal models of chronic neurodegeneration, such superimposed inflammatory activation can produce *de novo* pathology and has been shown to exacerbate the progression of neurodegenerative disease (Sheng et al., 2003, Kitazawa et al., 2005, Lee et al., 2008, Cunningham et al., 2009, Field et al., 2010, Krstic et al., 2012). However the mechanisms by which systemic inflammation exacerbate neurodegeneration remain unclear.

While many systemic inflammatory molecules may exacerbate cognitive decline, the role of systemic TNF-α has been of particular interest. It has been shown in a clinical population of AD patients that Systemic Inflammatory Events (SIEs) were associated with more rapid cognitive decline over 6 months (approximately 2-fold) and when those patients with SIE also had elevated plasma TNF-α levels, this was associated with a 10-fold greater rate of cognitive decline over the 6 month observation period (Holmes et al., 2009). Analysis of the Neuropsychiatric inventory (NPI) from the same cohort showed that elevated systemic TNF-α was associated with a 2-fold increase in symptoms characteristic of sickness behavior including apathy, anxiety, depression and agitation (Holmes et al., 2011). Elevated baseline TNF-α has also been associated with lower hippocampal volume (Sudheimer et al., 2014) and with greater likelihood of mild cognitive impairment (MCI) patient conversion to AD (Tarkowski et al., 2003) suggesting increased systemic TNF-α may have roles in hippocampal neurodegeneration. Neuroscientific studies of TNF-α have revealed a potentially complex biphasic role: administration of anti-TNF-α antibodies i.c.v. decreased Aβ, tau hyper-phosphorylation and memory deficits in AD-related models (Medeiros et al., 2007, Shi et al., 2011) but crossing of 3xTgAD mice with TNFR1/R2 double knockout mice actually exacerbated amyloid and Tau pathology and microglia from these mice had impaired phagocytic ability (Montgomery et al., 2011).

We sought to specifically investigate whether exogenously added systemic TNF-α has deleterious effects on cognitive function, inflammation, sickness behavior and neurodegeneration on a background of robust chronic neurodegeneration. In this study we used the ME7 model of prion disease, which shows robust synaptic loss, extracellular amyloidosis and a sequence of behavioral and cognitive impairments (Betmouni et al., 1999, Cunningham et al., 2003) and which we have previously shown to show acute neuronal death and more rapid decline after systemic challenge with LPS (Cunningham et al., 2005, Cunningham et al., 2009). We assessed the cognitive-disrupting effects of acute systemic TNF-α in ME7 and normal brain homogenate-injected controls (NBH) using a T-maze working memory test. We then examined hippocampal and hypothalamic mRNA, plasma proteins and hippocampal IL-1β protein induced by systemic TNF-α. The interaction of systemic TNF-α with existing pathology was also measured in ME7 and NBH animals using standard measures of sickness behavior: core body temperature, weight loss and open field activity. Finally the occurrence of *de novo* neuronal pathology after a single (i.p.) challenge with TNF-α was explored using quantification of apoptotic cell death, synaptic loss and tau hyperphosphorylation post-TNF-α in ME7 and NBH animals.

## 2 Methods

### 2.1 Animals and stereotaxic surgery

Female C57BL/6 mice (Harlan, Bicester, United Kingdom) were housed in cages of 5 at 21 °C with a 12:12 light, dark cycle. Food and water access was *ad libitum*. Mice were anaesthetized intraperitoneally (i.p.) with Avertin (2,2,2-tribromoethanol 50% w/v in tertiary amyl alcohol, diluted 1:40 in H_2_O; 20 ml/kg, i.p.; Sigma) and positioned in a stereotaxic frame (Kopf instruments). Holes were drilled at −2.0 mm (anteroposterior) and ±1.6 mm (lateral, either side of the midline) from Bregma and 1 μl 10% w/v ME7-infected C57BL/6 brain homogenate was injected into the hippocampus using a Hamilton microsyringe (Reno, Nevada, USA) to a depth of −1.7 mm. The needle was left in situ for 2 min before slow withdrawal. Control animals were administered 1 μl 10% w/v normal brain homogenate (NBH). Mice were placed in an incubator at ∼25 °C for recovery. When returned to their home cage they were administered Sucrose (5% w/v) and Carprofen (0.05% v/v; Rimadyl, Pfizer, Ireland) in their drinking water for 2 days post-surgery. Animals were monitored for recovery from surgery. All animal experimentation was performed under a license granted by the Minister for Health and Children, Ireland, with approval from the TCD Animal Research Ethics Committee and in compliance with the Cruelty to Animals Act, 1876 and the European Community Directive, 86/609/EEC. Every effort was made to minimize stress to the animals.

Animals were intraperitoneally administered recombinant mouse TNF-α (Peprotech, Rocky Hill, NJ, USA) at doses of 50 μg/kg or 250 μg/kg prepared in sterile saline (Sigma, Poole, UK). Lower doses were used for the T-maze experiments and higher doses for the pathology experiment. Our previous studies using these 2 TNF doses (Skelly et al., 2013) suggested that sickness at the earliest times points assessed was not terribly different between these 2 doses. Therefore we performed our cognitive experiments with the minimum dose that was practical (50 μg/kg i.p.) since more robust sickness may suppress the mouse behavioral repertoire too significantly to allow robust assessment of cognitive function (due to motivational and motor confounders). However, it has been our experience with LPS effects on cognition and *de novo* pathology that more intense systemic inflammation was required to produce damage (500 μg/kg LPS; (Cunningham et al., 2005)) versus that required to produce measureable cognitive dysfunction (100 μg/kg; (Murray et al., 2012)). This five-fold difference in potency guided our choice of doses in the current experiments with TNF-α. Control animals were injected using sterile saline (Sigma, Poole, UK) 200 μl per 20 g body weight. In additional experiments ME7 animals were challenged with LPS (Salmonella equine abortus, 500 μg/kg, Sigma, Poole, UK) in the presence or absence of the dominant negative TNF inhibitor XPRO1595 (a generous gift from David Szymkowski, Xencor, Monrovia, CA) at 30 mg/kg, i.p. 1 h before treatment with LPS. Appropriate action of XPRO1595 was assessed by administration of XPRO1595 (30 mg/kg i.p.) 1 h before TNF-α (50 µg/kg) to block TNF-induced hypothermia.

These TNF-α challenges were made at 16 weeks for T-maze experiments since we have previously established that ME7 animals at 18 weeks can no longer perform the T-maze at baseline (ie before acute inflammatory challenge). However our prior demonstrations of *de novo* neuropathology induced by systemic inflammation in ME7 animals has been exclusively at 18–19 weeks, which is the period when neuronal cell soma loss is determined to be occurring in this prion model (Cunningham et al., 2003, Cunningham et al., 2005).

### 2.2 Working memory

Hippocampal-dependent working memory was assessed in mice by observing alternation behavior in a novel water T-maze task (Murray et al., 2012). The T-maze, constructed from black perspex, was of the following dimensions (cm); long axis 67, short axis 38, depth 20 and arm width 7. Single 4 cm diameter holes were at the end of each choice arm, 2 cm from the floor and black exit tubes were fitted into these holes. Solid or permissive exit tubes could be fitted into these holes as appropriate, to facilitate/block escape at any location. A “guillotine” door was inserted to prevent access to one or other choice arm on the first run of each trial. Water to a depth of 2 cm at a temperature of 20 °C was poured into the maze; at this height a mouse must paddle and thus is motivated to leave the maze through an exit tube. Animals were taken with their cage mates to a holding cage. Each mouse was individually placed in the start arm of the maze with 1 arm blocked, such that they were forced to make a left or right turn. The arm sequences were chosen in a pseudo-random manner, with equal numbers of left and right turns in any one session; moreover, there were no more than 2 consecutive runs to the same arm. On making the turn the mouse could escape from the water by entering the exit tube and walking to a transit tube, in which it was carried to a new holding cage. The animal remained in this holding cage for a 30 s intra-trial interval, in which the guillotine door was removed and exit tube switched to the opposite arm. The mouse was then replaced in the start arm and could choose either arm. Since the exit tube which it had escaped through was now blocked, the mouse must alternate from its original turn to escape. On choosing correctly the mouse escaped to the transit tube and was returned to its home cage. On choosing incorrectly the mouse was allowed to self-correct to the correct exit arm. Animals were typically trained for >10 blocks of 10 trials (inter-trial interval of 20 min) before other experimental manipulations are performed. Most animals reached baseline alternation of greater than 80%. No animals were experimentally challenged unless they performed at 70% or above for consecutive days. Furthermore, no animals that performed lower than 80% in the last block of 5 trials or displayed evidence of a side preference on the day prior to experimental challenges were included. Thereafter, animals were challenged with saline or TNF-α (50 μg/kg) and then tested in the T-maze for 15 trials across 6 h (1–3 h, 3–5 h and 5–7 h) and again at one day later to assess recovery.

### 2.3 Sickness behavior

An ‘open field’ consisted of a plastic base (58 cm × 33 cm) surrounded by walls of 19 cm. The floor of the box is divided into a grid of equal sized squares. Measurement was made of distance travelled (grid squares crossed) and total number of rears (frequency with which the mice stood on their hind legs in the maze). Each animal’s baseline activity was measured prior to experimentation. Animals were scored for 2 min in the Open Field.

A thermocouple rectal probe (Thermalert TH5, Physitemp; Clifton, NJ, USA) was used to measure core body temperature. The mice were habituated to measurement of rectal temperature for 2 days prior to experimentation to minimize/standardise stress effects. Baseline temperature was taken at the time of challenge, followed by the timepoints indicated. Body weight was likewise taken immediately before challenge and at a number of times up to 18 h (the time point at which these animals were euthanised and perfused for histopathology).

### 2.4 Tissue preparation

Animals for mRNA and protein analysis were terminally anaesthetised with sodium pentobarbital at 2 or 4 h post-challenge (Euthatal, Merial Animal Health, Essex, UK). The thoracic cavity was opened and blood collected in heparinised tubes directly from the right atrium of the heart. The animal was then rapidly transcardially perfused with heparinised saline to wash out the blood. Whole blood was spun at 1500x*g* for 15 min to remove cells and the plasma was then aliquoted and stored at −20 °C. Three tissue punches were taken from the brain: the left hippocampus, right hippocampus and the hypothalamus at the appropriate anterior-posterior coordinates from Bregma. Tissue was snap-frozen in liquid nitrogen and was stored at −80 °C until use.

Animals for immunohistochemical examination were terminally anaesthetised with sodium pentobarbital (Euthatal, Merial Animal Health, Essex, UK) at 9 or 18 h post-challenge and transcardially perfused with heparinised saline for approximately 3 min followed by 10% neutral buffered formalin (Sigma, Poole, UK) for approximately 15 min. Brains were post-fixed in formalin and then embedded in paraffin wax. Coronal sections (10 μm) were cut on a Leica RM2235 Rotary Microtome (Leica Microsystems, Wetzlar, Germany) at the level of the hippocampus and floated onto electrostatically charged slides (Menzel-Glaser, Braunschweig, Germany) and dried at 37 °C overnight.

### 2.5 Plasma ELISA assays

Plasma samples were diluted appropriately and then analysed for TNF-α and CCL2. Mouse TNF-α and CCL2 were quantified by sandwich-type ELISA, using R&D systems duo set kits (R&D systems, Minneapolis, MN, USA). A standard protocol was followed. The capture anti-TNF-α and CCL2 antibodies were diluted 1/180 (approximately 1 μg/ml) in PBS, and used to coat a 96-well maxisorb microplate overnight (Nunc, Fisher Scientific, Leicestershire) with 100 μl per well. Plates were then washed with PBS + 0.05% Tween and blocked with PBS + 1% BSA before addition of 100 μl samples and standards for 2 h at room temperature. Detection antibodies were used at a dilution of 1/180 and incubated on the plates for 1.5 h. Streptavidin poly-horseradish peroxidase (HRP: Sanquin, Amsterdam, Netherlands) was diluted in 1:10,000 and incubated in the dark at room temperature for 20 min. Samples, standards, detection antibodies and streptavidin poly-horseradish peroxidase were diluted in high performance ELISA buffer (Sanquin; Amsterdam, Netherlands). TMB and H_2_O_2_ were used as substrate and the reaction was stopped with 1 M H_2_SO_4_ before optical density was read at 450 nm with correction at 570 nm. Standard curves were constructed and samples were quantified only if the absorbance fell on the linear portion of the standard curve. IL-1β protein in the hippocampus was quantified using a Quantikine mouse IL-1β kit (R&D systems, Minneapolis, MN, USA) according to manufacturer’s instructions.

### 2.6 RNA extraction and quantitative PCR

Total RNA was isolated using the RNeasy Plus Mini method (Qiagen, Limburg, Netherlands) as per the manufacturer’s instructions. To ensure complete DNA elimination from the column-bound RNA, an on-column DNase step was performed. The RNA yield and quality of each sample was quantified based on Optical Density (OD) using the NanoDrop’ND-1000 UV–vis spectrophotometer (Thermo Fisher Scientific, Dublin, Ireland). cDNA synthesis was carried out using a High Capacity cDNA Reverse Transcriptase Kit (Applied Biosystems, Warrington, UK). cDNA was stored at −20 °C until use in RT-PCR. All primer and probe sets were designed using Applied Biosystems Primer Express software and amplified a single sequence of the correct amplicon size, as verified by SDS-PAGE. Where no probe sequence is shown, the DNA binding dye SYBR green was used in its place. Primer pair/probe sequences are shown in Table 1. Samples for RT-PCR were run in duplicate and contained 12.5 μl Faststart universal probe master mix (Roche, Lewes, UK), 0.5 μl of each of the forward primer (10 μM), reverse primer (10 μM) and probe (10 μM), and 10 μl RNase-free water. All PCR was carried out in a StepOne Real-Time PCR system (Applied Biosystems, Warrington, UK) under the cycling conditions: 95 °C for 10 min followed by 95 °C for 10 s and 60 °C for 30 s for 40 cycles. Quantification was achieved by exploiting the relative quantitation method, using cDNA from LPS-injected mouse brain as a standard expressing all genes of interest and fourfold serial dilutions of this cDNA to construct a linear standard curve relating cycle threshold (CT) values to relative concentrations. This method has been described in detail elsewhere (Cunningham et al., 2005). Gene expression data were normalized to the housekeeping gene glyceraldehyde-3-phosphate dehydrogenase (GAPDH).

### 2.7 Immunohistochemistry

Sections were labelled with IBA-1, 1/2000, ab5076 (Abcam, Cambridge, UK), SY38, 1/2000 (Millipore, Cork, Ireland), AT8, 1/200, MN1020 (Thermo Scientific, MA, USA), Caspase 3, 1/50, ab3623 (Millipore, Cork, Ireland); SY38 sections were treated with 0.2 M boric acid, pH 9 at 65 °C for 30 min then cooled to room temperature prior to quenching. All sections were quenched with 1% H_2_O_2_/methanol (20 min). Sections were then pre-treated with citrate buffer (pH 6) for 2 × 5 min in the microwave (except SY38). IBA-1 sections were then pre-treated with 0.04% pepsin in 0.1 M HCl for 20 min prior to blocking. Slides were blocked with the appropriate serum. Primary antibodies were incubated overnight at 4 °C. Thereafter the ABC method was used as previously described (Cunningham et al., 2005) with peroxidase as enzyme, 3,3′-Diaminobenzidine as chromogen and H_2_O_2_ as substrate. Slides were counterstained using Haemotoxylin (VWR International Ltd, Dublin, Ireland). The DeadEnd Fluorometric TUNEL system (G3250, Promega) was used in conjunction with a biotinylated anti-Fluorescein secondary to visualise apoptotic cells in accordance with manufacturer’s instructions.

### 2.8 Immunohistochemical quantification

TUNEL-positive and Caspase-3-positive cells were counted by two experimentors, blinded to experimental treatment. Tau was quantified in the CA3 region by determining the area fraction labelled by the AT8 antibody. The density of synaptophysin staining was determined by monochrome pixel density analysis of digitally captured images using Leica QWIN image analysis software (Leica, Milton Keynes, UK) as described in (Cunningham et al., 2003). The transmittance of the stratum radiatum was expressed as a ratio to the transmittance in the stratum lacunosum (a layer relatively low in staining, to achieve a contrast) or as a ratio to the mossy fibre layer (a layer maintaining reasonably dark staining throughout the progression of disease) as follows:

$$\text{Ratio}=({\text{T}}_{\text{dentate gyrus}}-{\text{T}}_{\text{radiatum}})/({\text{T}}_{\text{dentate gyrus}}-{\text{T}}_{\text{lacunosum}})$$

IBA-1 quantification: The number of Iba-1 positive cells was determined, in 10 μm sections, in the dorsal part of the hippocampus (between dentate gyrus granule and CA1 pyramidal layers) in high-quality microphotographs taken under 20X objective. At least four counting frames per animal were analysed (equal left and right hemisphere) with Image J software (NIH, Bethesda, MD, USA). Files were converted to 8 bit binary images and the intensity threshold set at 150. The number of cells per field (0.280 mm^2^) was automatically determined by counting all the objects between 200 and 6000 pixels^2^.

### 2.9 Statistics

Behavioral data were analysed using 3-way ANOVA (with disease and treatment as between subjects factors and time as a repeated measures, within subjects, factor) followed by Bonferroni *post-hoc* pair-wise comparisons where appropriate. Molecular data were analysed using 2-way ANOVA followed by Bonferroni *post-hoc* tests. Histological counts were analysed using one-way ANOVA followed by Dunnett’s multiple comparison.

## 3 Results

### 3.1 Cognitive deficits following systemic TNF-α

**Fig. 1:**
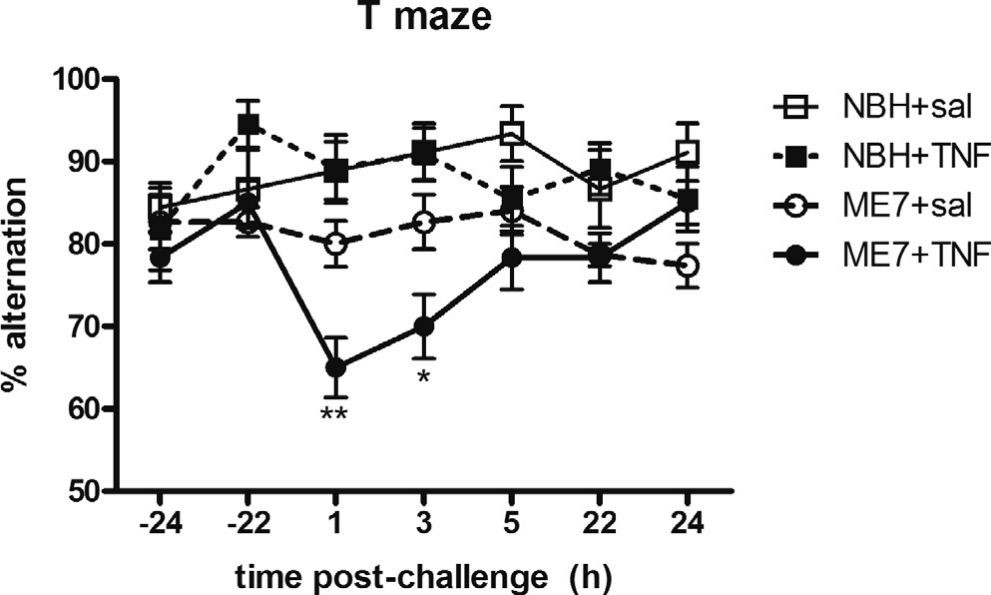
Impact of systemic TNF-α on working memory in NBH and ME7 animals. Percentage T-maze alternation post-systemic TNF-α (50 μg/kg) or saline administration in ME7 or NBH animals, 16 weeks post-inoculation. Data were analysed using a two-way ANOVA followed by a Bonferroni multiple comparison test comparing individual time-points. Three-way ANOVA was used to test overall significance. Statistically significant differences are denoted by ^∗^(P < 0.05), ^∗∗^(P < 0.01), ^∗∗∗^(P < 0.001). All data are represented as the mean ± SEM, n = 9–15 for all groups.

The effect of TNF-α (50 μg/kg) on working memory was examined using the alternation T-maze. All animals were confirmed to have acceptable baseline performance ⩾70% for three consecutive days pre-challenge. On the days of saline or TNF-α treatments, animals underwent 3 blocks of 5 trials (between 1 and 7 h post-challenge) and 2 further blocks of 5 trials at 24-h post-challenge to confirm recovery. Systemically administered TNF-α induced a transient deficit in working memory in the T-maze task in ME7 animals, while there were no deficits in NBH animals similarly challenged (Fig. 1). Using 3-way repeated measures ANOVA there was an interaction between disease, treatment and time following systemic TNF-α challenge (P < 0.01, F_6,328_ = 3.09). An *a priori* prediction from previous studies in the laboratory suggested that the effect of TNF-α on working memory would be a transient one (Fig. 1) and at the one hour (P < 0.01) and three hour (P < 0.05) timepoints following systemic TNF-α, ME7 + TNF-α animals were significantly different from all other treatment groups. Therefore, prior neurodegeneration in the ME7 model is a risk factor for acute cognitive deficits following acute systemic elevation of TNF-α.

### 3.2 Systemic TNF-α induces heightened CNS inflammatory response

Transcripts for selected inflammatory cytokines were examined in the hippocampus and hypothalamus following systemic TNF-α administration. IL-1β, TNF-α and the chemokine CCL2 all showed acute elevation after systemic TNF-α that was greater in ME7 animals than in NBH.

**Fig. 2:**
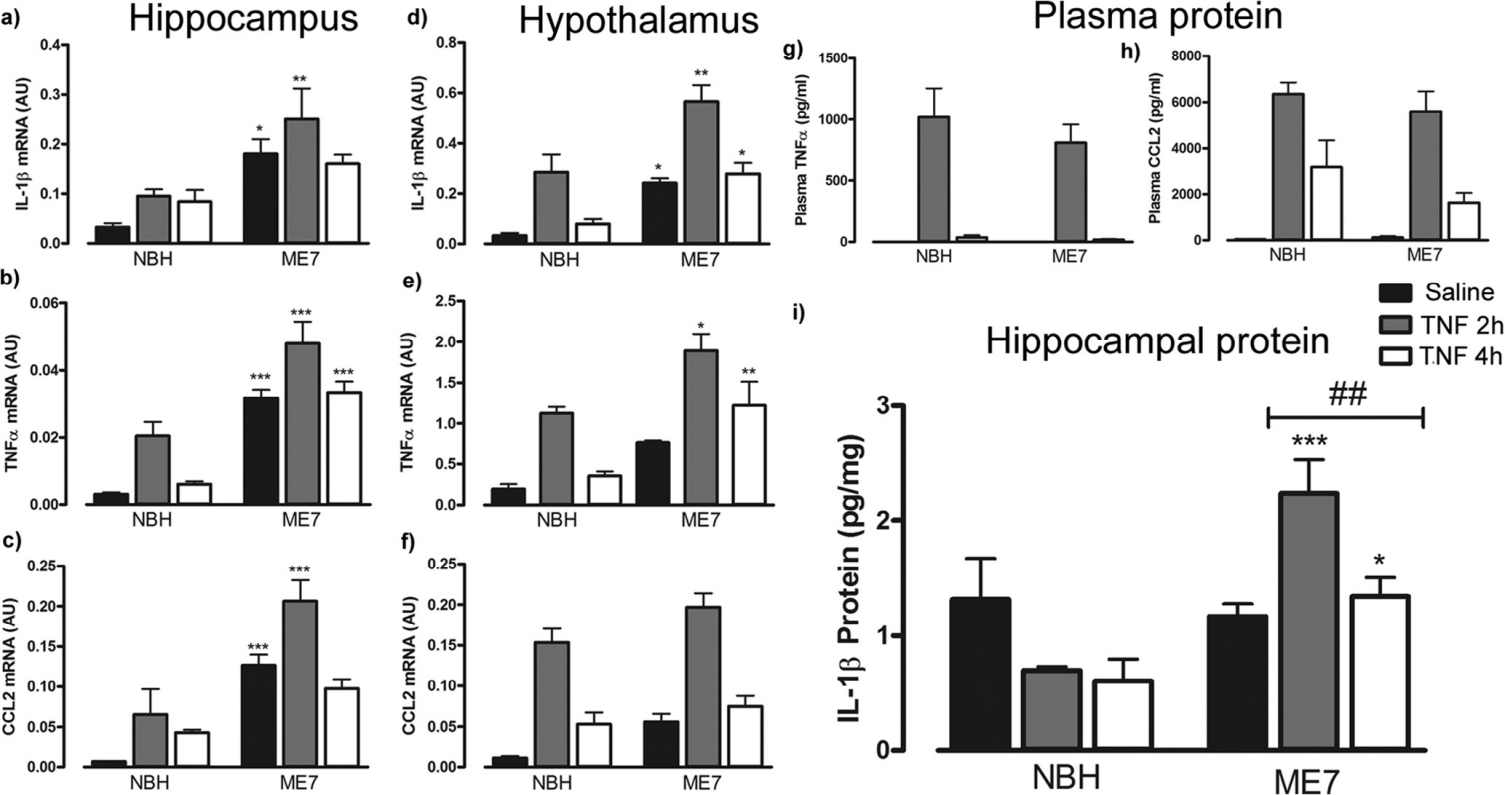
Central and systemic inflammatory markers post-systemic TNF-α. Hippocampal (a-c) and hypothalamic (d-f) transcripts, and plasma (g-h) and hippocampal (i) protein at 2 and 4 h post TNF-α (50 μg/kg) i.p in ME7 or NBH animals, 16 weeks post-inoculation. Data were analysed using a two-way ANOVA followed by Bonferroni *post-hoc* test. Statistically significant differences between NBH and ME7 groups are denoted by ^∗^(P < 0.05), ^∗∗^(P < 0.01), ^∗∗∗^(P < 0.001). # denotes a disease × treatment interaction. All data are represented as the mean ± SEM, n = 3–5 for all groups.

IL-1β mRNA expression revealed a significant effect of disease [F_(1,18)_ = 24.66, P < 0.001]. *Post-hoc* Bonferroni comparisons revealed a significant difference between NBH + TNF-α and ME7 + TNF-α at 2 h (P < 0.01) and between NBH + saline and ME7 + saline (P < 0.05) (Fig.2a). Disease [F_(1,21)_ = 72.99, P < 0.001], and TNF-α administration [F_(2,21)_ = 10.97, P < 0.001] both significantly increased hippocampal TNF-α expression. *Post-hoc* comparisons showed that disease was associated with significantly (p < 0.01) higher hippocampal TNF-α mRNA expression across all treatment groups (P < 0.001) (Fig.2b). CCL2 mRNA expression in the hippocampus following peripheral TNF-α administration revealed a significant effect of disease [F_(1,18)_ = 49.15, P < 0.001], and of TNF-α [F_(2,18)_ = 9.03, P < 0.01]. *Post-hoc* Bonferroni comparisons revealed significant differences between NBH + TNF-α at 2 h and ME7 TNF-α at 2 h (P < 0.001) but also between NBH + saline and ME7 + saline (P < 0.001) (Fig.2c).

The same inflammatory genes were examined in the hypothalamus following TNF-α administration. IL-1β mRNA showed a significant effect of disease [F_(1,20)_ = 32.53, P < 0.0001], and of TNF-α treatment [F_(2,20)_ = 20.03, P < 0.0001], but no significant disease × treatment interaction. *Post-hoc* Bonferroni comparison revealed a significant increase in IL-1β mRNA expression in the hypothalamus in all ME7 groups compared to all NBH groups (Fig.2d): saline (P < 0.05), TNFα-2 h (P < 0.01) and TNF-α 4 h (P < 0.05). TNF-α mRNA expression in the hypothalamus revealed a significant effect of disease [F_(1,20)_ = 28.47, P < 0.0001] and of TNF-α treatment [F_(2,20)_ = 19.5 P < 0.0001] but there was no disease × treatment interaction (Fig.2e). Nonetheless, *post-hoc* Bonferroni comparison revealed a significant increase in TNF-α mRNA expression in ME7 treated with TNF-α at 2 h (P < 0.05) and 4 h (P < 0.01) in comparison to the same treatments in NBH animals. CCL2 mRNA in the hypothalamus revealed a significant effect of disease [F_(1,20)_ = 10.53, P < 0.01] and TNF-α treatment [F_(2,20)_ = 58.34, P < 0001]. In the case of CCL2, *post-hoc* Bonferroni comparison revealed no significant difference between NBH + TNF-α and ME7 + TNF-α groups in the hypothalamus at 2 h post-challenge (Fig.2f).

Systemic levels of TNF-α and CCL2 proteins were examined in the plasma using ELISA (Fig. 2g,h). TNF-α treatment predictably significantly increased serum TNF-α protein levels [F_(2,24)_ = 45.67777, P < 0.0001]. CCL2 also showed a significant increase in plasma following TNF-α treatment [F_(2,21)_ = 11.71, P < 0.001] but levels of both of these cytokines were equivalent in TNF-α-challenged ME7 and NBH animals.

Hippocampal expression of IL-1β protein was examined using ELISA following systemic TNF-α administration (Fig.2i). IL-1β protein in the hippocampus was modestly increased by disease [F_(1,19)_ = 19.8, P < 0.001] and by TNF-α treatment [F_(2,19)_ = 3.53, P < 0.05]. However, there was also a significant disease × treatment interaction [F_(2,19)_ = 8.947, P < 0.001] and *post-hoc* Bonferroni comparison revealed a significant increase in hippocampal IL-1β protein in ME7 animals compared to NBH animals at both 2 (P < 0.001) and 4 (P < 0.05) hours post-TNF-α.

**Fig. 3:**
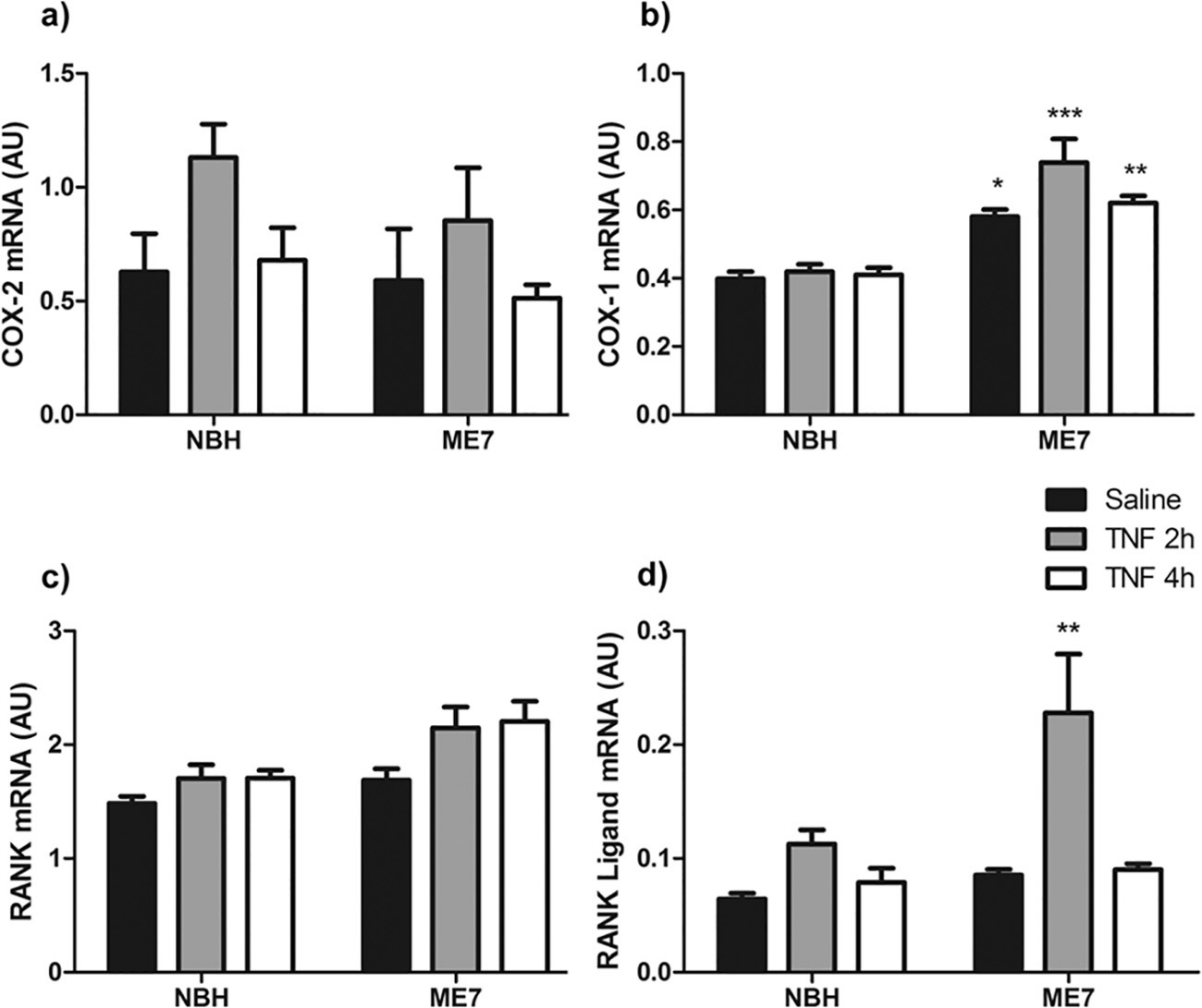
Expression of genes implicated in hypothalamic thermoregulatory activity. Changes in the mRNA expression of COX-2 (a) COX-1 (b) receptor activator of nuclear factor κB (RANK) (c) and receptor activator of nuclear factor κB ligand (RANK Ligand) (d) after i.p. challenge with non-pyrogenic saline or 2 or 4 h after systemic TNF-α treatment (50 μg/kg) in NBH and ME7 animals. Data were analysed using two way ANOVA followed by Bonferroni *post-hoc* test. Statistical significance is denoted by ^∗^(P < 0.05), ^∗∗^(P < 0.01), ^∗∗∗^(P < 0.001). All data are represented as the mean ± SEM, n = 4–5 for all groups.

To further explore the brain inflammatory milieu, the hypothalamic mRNA expression of other genes implicated in sickness behavior and thermoregulation were assessed (Fig. 3). COX-2 mRNA expression was significantly increased by TNF-α treatment at 2 h, with this increase declining by 4 h (Significant effect of treatment [F_(2,21)_ = 3.624, P < 0.05]; Fig.3a). TNF-α did not have different effects in NBH and ME7 animals.

COX-1 was not significantly altered by TNF-α treatment but was significantly increased in diseased versus normal animals. Two way ANOVA revealed a significant effect of disease [F_(1,21)_ = 60.76, P < 0.0001] and *post-hoc* Bonferroni comparisons showed a significant difference between NBH + saline and ME7 + saline (P < 0.05) and between NBH + TNF-α and ME7 + TNF-α at 2 h (P < 0.001) and at 4 h (P < 0.01) (Fig.3b).

Receptor activator of nuclear factor κB (RANK) expression was slightly increased by TNF-α treatment and slightly increased by disease per se. Both disease [F_(1,21)_ = 11.10, P < 0.005] and treatment [F_(2,21)_ = 4.04, P < 0.05] showed significant effects, albeit with a small effect size.

Regarding RANK ligand (RANKL), this was robustly induced by TNF-α, particularly in ME7 animals. Two way ANOVA showed a significant effect of disease [F_(1,21)_ = 5.65, P < 0.05] and treatment [F_(2,21)_ = 9.072, P < 0.005]. Although the interaction was not quite significant [F(2,21) = 2.75] Bonferroni *post-hoc* test revealed a significant increase in ME7 + TNF-α at 2 h when compared with NBH + TNF-α at 2 h (P < 0.01) (Fig.3d).

### 3.3 TNF-α induces an exaggerated sickness response in ME7 animals

**Fig. 4:**
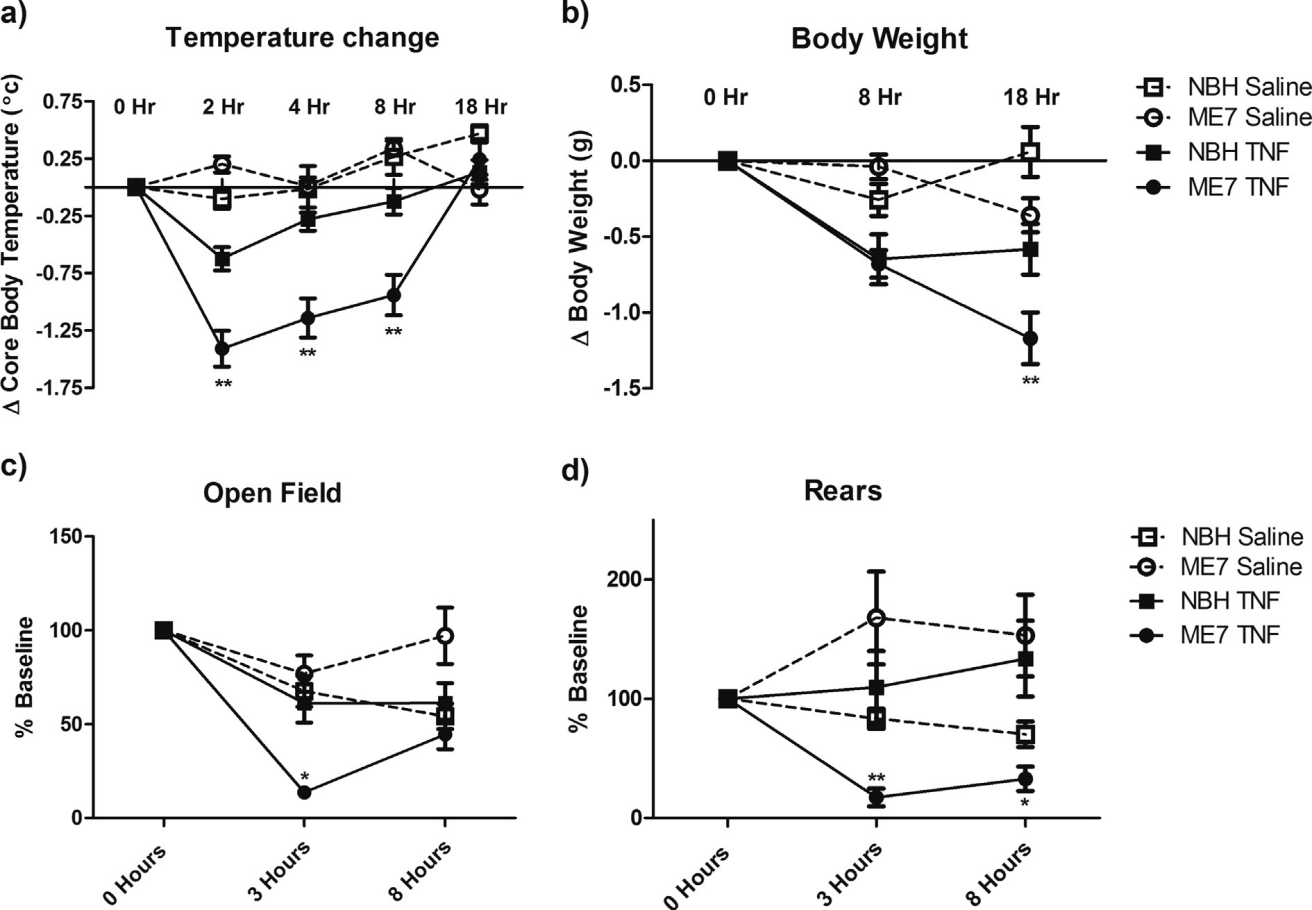
Impact of systemic TNF-α on sickness behavior in NBH and ME7 animals. Sickness behavior was examined following TNF-α (250 μg/kg) i.p. administration in ME7 prion diseased mice 18 weeks post-inoculation. Temperature change and body weight are presented as change from baseline. Rears and open field are graphed as percentage of baseline. Data were analysed using a three-way ANOVA followed by Bonferroni multiple comparison test comparing each treatment group to its relevant control group. Statistical significance is denoted by ^∗^(P < 0.05), ^∗∗^(P < 0.01), ^∗∗∗^(P < 0.001). All data are represented as the mean ± SEM, n = 6–15 for all groups.

The data on CNS cytokines and RANKL predicted differential effects of systemic TNF-α (250 μg/kg) on sickness parameters in NBH and ME7 animals. Therefore core body temperature, body weight, locomotor activity and rears in the open field were examined at the indicated times post-challenge. TNF-α induced acute hypothermia peaking at 2 h and returning gradually to baseline at 18 h (Fig.4a). The reduction in core body temperature was significantly more severe in ME7 animals. There was a main effect of TNF-α (P < 0.001, F_(1,174)_ = 19.91) and an interaction between disease, treatment and time (P < 0.001, F_(4,174)_ = 15.94). TNF-α caused modest weight loss, presumably by inducing acute hypophagia following treatment (P < 0.0001, F_(1,104)_ = 34.36) and this was also somewhat greater in ME7 + TNF-α animals, when measured at 18 h (Fig.4b). There was an interaction between treatment and time (P < 0.0001, F_(2,104)_ = 17.63).

TNF-α challenge appeared to reduce levels of activity in the open field (Fig.4c) but this was only evident in the ME7 + TNF-α group (Fig.4c), showing a particularly robust decrease in activity at 3 h (P < 0.001). There was a main effect of TNF-α (P < 0.01, F_(1,139)_ = 8.58) and an interaction between disease, treatment and time (P < 0.01, F_(3,139)_ = 4.67). There was no main effect of treatment on rears however, there was a robust interaction between disease, treatment and time (P < 0.001, F_(3,139)_ = 11.06). The ME7 + TNF-α group once again showed a prolonged decrease in rearing activity following systemic TNF-α treatment (Fig.4d) while no such decrease was observed with NBH + TNF-α animals. Thus TNF-α has significantly greater activity suppression in animals with prior degeneration than in normal animals.

### 3.4 A single i.p. challenge with TNF-α does not exacerbate ME7 pathology

**Fig. 5:**
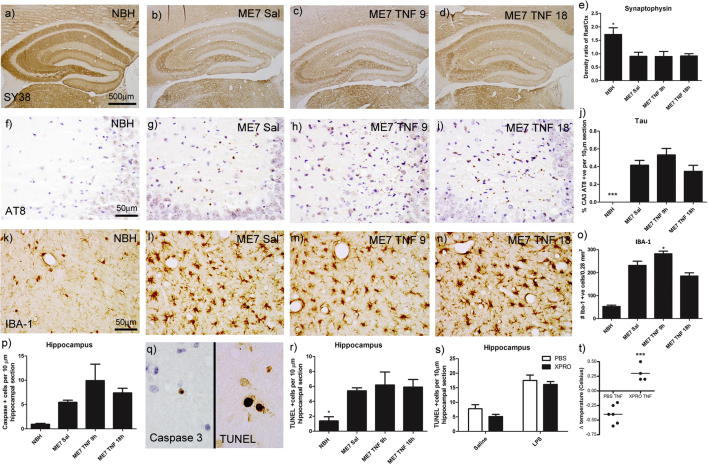
Immunohistochemical analysis of systemic TNF-α effects on prior pathology. Pathological examination was performed 18 h post-TNF-α (250 μg/kg) i.p in ME7 prion diseased mice 18 weeks post-inoculation. Labelling and quantification was performed for Synaptophysin × 5 (a–d, e), hyperphosphorylated Tau × 40 (f–i, j), the microglial marker IBA-1 × 40 (k-n, o), and apoptosis markers Caspase 3 × 100 (p, q) and TUNEL x 100 (q, r). Data were analysed using a one-way ANOVA followed by a Dunnett’s multiple comparison test. Statistical significance is denoted by ^∗^(P < 0.05), ^∗∗∗^(P < 0.001). All data are represented as the mean ± SEM, n = 3–5 for all groups. Additional hippocampal analysis was performed in ME7 animals 18 weeks post-inoculation; 18 h post i.p. saline or LPS (500 μg/kg). Animals were pretreated with either PBS or XPRO1595 (30 mg/kg). TUNEL positive cells were quantified in hippocampus (s) and data were analysed using two-way ANOVA followed by a Bonferroni multiple comparison test. Appropriate action of XPRO1595 (30 mg/kg) was verified by inhibition of TNF-α-induced (50 μg/kg) hypothermia in normal C57BL/6 mice (t). All data are represented as the mean ± SEM, n = 3–6 for all groups.

The effect of a single systemic challenge with TNF-α (250 μg/kg) on ME7 pathology was examined using markers for microglial activation and neuronal and synaptic pathology (synaptophysin, Tau, IBA-1, activated caspase 3 and TUNEL). Synaptophysin was significantly decreased in the hippocampal layers by ongoing disease pathology in ME7 in comparison to NBH (Fig.5a vs. b). However, this was not further decreased 9 or 18 h post-systemic TNF-α (Fig.5a–d, e). There was significant deposition of Hyperphosphorylated Tau throughout coronal sections from ME7 animals with significant concentration in the CA3 region (Fig.5f–i). This was not altered by administration of systemic TNF-α (Fig.5j). Microgliosis was evident with the ongoing ME7 pathology and the major difference was between NBH animals and all ME7 groups (Fig. 5k–n, m). However there was an apparent moderate increase in IBA-1 positive cells at 9 h post-TNF, which was not apparent at 18 h post-TNF. After a significant one-way ANOVA, ME7 + TNF at 9 h was significantly different to both ME7 + saline (p < 0.05) and ME7 + TNF 18 h (p < 0.001).

Both caspase 3 and TUNEL were used to determine whether systemic TNF-α could increase ongoing apoptosis in the ME7 model (examples in Fig.5q). In all regions counted in ME7 animals there was a significant level of ongoing apoptosis, as shown by increased numbers of activated caspase 3-positive cells in the ME7 groups (Fig.5p, q). However TNF-α did not induce further statistically significant increases in the numbers of these cells.

Terminal deoxynucleotidyl transferase dUTP nick end labelling (TUNEL) was also used to visualise apoptosis since it labels fragmentation of nuclear DNA. TUNEL-positive cells appeared throughout coronal sections of ME7 brains, appearing as dense labelling within a condensed nucleus. In the hippocampus the ME7 + Saline group significantly greater levels of apoptosis were apparent compared to the NBH animals (P < 0.05; Fig.5r) but the number of TUNEL-positive apoptotic cells was not significantly increased by administration of systemic TNF-α, suggesting that acutely elevated TNF-α does not, of itself, significantly increase hippocampal cell death even in the vulnerable brain. We had previously shown that systemic LPS (500 μg/kg) acutely increases the number of TUNEL-positive cells in the hippocampus (Cunningham et al., 2005) and we thus interrogated whether blocking systemic TNF-α was sufficient to protect against this acute cell death. TUNEL-positive cells were quantified in the hippocampus of ME7 animals 18 weeks post-inoculation; 18 h post i.p. challenge with saline or LPS (500 μg/kg), having been pretreated with either PBS or the dominant negative TNF inhibitor XPRO1595 (30 mg/kg). As previously reported, systemic LPS induced a greater level of apoptosis in the hippocampus (P < 0.0001, F_(1,19)_ = 48.23) of ME7 animals (Fig.5s) but this was not mitigated by pre-treatment with XPRO1595. As a positive control to demonstrate appropriate blockade of TNF-α action by XPRO1595, mild TNF-α-induced hypothermia was abrogated by XPRO1595 treatment (30 mg/kg) 60 min prior to TNF-α (50 μg/kg; Fig.5t).

## 4 Discussion

In the current study we have demonstrated that acutely elevated systemic TNF-α is sufficient to induce acute cognitive dysfunction and exaggerated sickness behavior and this occurs selectively in animals with existing progressive neurodegeneration. Such a heightened response to equivalent systemic inflammation could be brought about by exaggerated CNS production of relevant pro-inflammatory mediators, for which we present some evidence. Although systemic TNF-α did further elevate the hippocampal and hypothalamic inflammation present in these degenerating brains, a single such challenge was not sufficient to induce measureable *de novo* neuropathological changes.

### 4.1 Acute cognitive dysfunction

The potential impacts of systemic inflammation on cognitive function have been widely discussed in the literature. The evidence that these effects are robust in normal healthy humans remains relatively limited despite multiple rodent studies suggesting such deficits (Cunningham and Sanderson, 2008, Yirmiya and Goshen, 2011, Czerniawski and Guzowski, 2014). Though some effects have been observed in humans these have been relatively mild or inconsistent (Reichenberg et al., 2001, Grigoleit et al., 2010) or noted on tasks that are relatively difficult (Harrison et al., 2014). The evidence that systemic inflammation has more robust effects on the frail or degenerating brain is accumulating in rodent studies (Barrientos et al., 2006, Buchanan et al., 2008, Field et al., 2012, Murray et al., 2012) and this is consistent with inflammatory triggering of human disorders such as delirium, which is a profound and acute cognitive impairment that frequently occurs during acute medical illness or after inflammatory traumas such as orthopedic fracture and surgery (Cunningham and Maclullich, 2013, Inouye et al., 2014). Existing cognitive impairment and neurodegenerative pathology are the key risk factors for delirium incidence and it has been shown that the risk of delirium is proportional to the extent of underlying pathology/baseline cognitive impairment (Davis et al., 2015). It is significant that in this model of acute systemic inflammation TNF-α alone is sufficient to induce acute dysfunction in a cognitive task reliant on attentional and working memory function, two key cognitive domains impaired in delirium. Associations between delirium and TNF-α in biomarker studies have sometimes (Adamis et al., 2011, Kazmierski et al., 2014), but not always (van den Boogaard et al., 2011, Ritter et al., 2014), been detected. The current data are certainly supportive of deleterious effects of systemic inflammation on short-term cognitive processes. When patients show post-surgical cognitive dysfunction but do not meet criteria for delirium they may be described as experiencing post-operative cognitive dysfunction (POCD). Although the terms delirium, sub-syndromal delirium and POCD are not always associated together, these probably represent a severity continuum of cognitive impairments after acute inflammatory/surgical trauma. A number of groups are now investigating cognitive consequences of tibial fracture in rodents as a model system for POCD. Memory consolidation deficits have most often been studied using contextual fear conditioning and it has been shown that tibial fracture-induced deficits in CFC could be blocked via systemic blockade of TNF-α (Terrando et al., 2010). This indicates that peripheral TNF-α may be a key acute injury-associated cytokine that triggers cognitive changes during such episodes. Those studies showed acute TNF-α-dependent memory consolidation deficits even in young healthy animals upon tibial fracture. The current study indicates that TNF-α alone is not sufficient to impair working memory when administered to normal animals but can impair these cognitive domains when applied to animals with existing neurodegenerative changes in the hippocampus, thalamus and basal forebrain (Cunningham et al., 2003, Davis et al., 2015). It is of interest that the studies of Terrando and colleagues showed that systemic TNF-α-induced memory consolidation deficits were mediated via hippocampal expression of IL-1β, the production of which was heightened in those animals with neurodegeneration in the current study. Elevated CSF levels of IL-1β have recently been shown to be associated with delirium in hip-fracture patients (Cape et al., 2014). The causative role of TNF-α in acute delirium and POCD, and potential involvement of brain IL-1β require investigation in humans.

### 4.2 Sickness Behavior and inflammatory mediator synthesis

TNF-α produced very marked hypothermia, weight loss and suppression of activity in animals with existing neurodegenerative disease, with respect to normal healthy animals similarly challenged. This cytokine, injected alone, has been reported to produce mild but measureable sickness behavior in healthy normal animals by ourselves and others (Bluthe et al., 1994, Swiergiel et al., 1997, Skelly et al., 2013, Biesmans et al., 2015) and sickness induced by other inflammatory stimuli such as bile-duct ligation (D’Mello et al., 2009) and CD40 ligation (Cathomas et al., 2015) are reported to be TNF-mediated. The sickness observed in normal animals in the current study was not particularly robust, with mild effects on core-body temperature and body weight and no clear decrease in open field activity or rears. The analysis of the inflammatory milieu (Fig. 2, Fig. 3) demonstrate elevation of multiple inflammatory markers, even at the lower doses used in these studies, and these are consistent with other studies of similar doses (Biesmans et al., 2015). It is possible that mild TNF-induced suppression of activity is masked by habituation to the open-field observed in saline-treated controls. Whatever the reasons for this relatively limited response in normal animals, the ME7 + TNF-α animals show marked hypothermia and robust hypoactivity by measures of distance and rears, despite similar levels of circulating cytokines, indicating exaggerated brain responses to equivalent systemic inflammation. We have already described exaggerated sickness responses of ME7 animals to LPS (Combrinck et al., 2002) and this inflammatory hypersensitivity has been observed also in aged animals (Godbout et al., 2005). Sickness behavior is not routinely measured in humans but it is of interest that elevated systemic TNF-α was associated with a 2-fold increase in neuropsychiatric features in AD patients, including apathy, anxiety, depression and agitation, which are also characteristic of sickness behavior (Holmes et al., 2011). Human volunteer studies with bacterial endotoxin show an association between plasma TNF-α and acute changes in anxiety (Lasselin et al., 2016). Our current data, when assessed alongside these human association studies, indicate that elevated TNF-α is likely to be causative in altering aspects of human cognitive and affective behavior.

It is important to point out that the levels of circulating TNF-α at the current doses (Skelly et al., 2013) are likely to be rather high compared to those observed in humans during acute illness, but are certainly comparable to those induced by experimental endotoxemia in humans (van den Boogaard et al., 2010). Moreover, at 50 μg/kg, TNF-α levels compare with those induced by low dose LPS (100 μg/kg i.p.) and, at 250 μg/kg, levels compare with those induced in mouse LPS (Pavlov et al., 2009) and cecal ligation and puncture (Osuchowski et al., 2006) models of sepsis. Balancing this, it is important to point out that these levels are rapidly cleared and sickness in our TNF-treated animals is clearly rather limited in NBH animals.

The hypothalamus is at the heart of many sickness-associated changes with specific and well-characterised roles in fever and anorexic responses (Dantzer et al., 2008, Saper et al., 2012). ME7 animals produce heightened levels of transcripts for hypothalamic pro-inflammatory mediators, including TNF-α, upon exposure to systemic TNF-α, compared to normal animals similarly challenged. Although these hypothalamic responses are not elevated in a truly exaggerated fashion, the heightened increase may be sufficient to explain the more robust hypothermia and weight loss observed in ME7 + TNF-α animals. However, we cannot rule out the possibility that neurons (in the hippocampus and/or hypothalamus) are hyper-responsive to TNF-α or mediators induced by TNF-α.

Indeed it is likely that at least some of the effects of systemic TNF-α are mediated by secondary inflammatory mediators. With respect to hypothermic effects, TNF-α is generally described as a cryogen (Leon, 2004) and has been shown to produce hypothermia in mice independent of IL-1 (Mannel et al., 1987). However, prostaglandins, synthesised by cyclo-oxygenases 1 and 2 are the major mediators of temperature and anorexic responses to LPS (Saper et al., 2012) and systemic TNF-α also induces COX-2 transcription and expression of COX-2 protein at the brain endothelium (Skelly et al., 2013). Our current data show TNF-induced COX-2 expression but this was not different in normal (NBH) and ME7 animals, indicating that exaggerated sickness responses are not underpinned by exaggerated COX-2 expression. It is conceivable that the disease-associated increase in COX-1 (Fig. 3), which is consistent with previous results in ME7 animals (Griffin et al., 2013), may make some additional contribution to changes such as hypothermia and hypoactivity, which have been shown to be robustly influenced by prostaglandins (Teeling et al., 2010, Saper et al., 2012).

A very significantly elevated expression of RANKL in TNF-treated ME7 animals may also contribute to the exaggerated hypothermia observed. RANK and RANK ligand belong to the TNF superfamily and they are critical for thermoregulation in the central nervous system (see Hanada et al., 2010 for review). TNF-α is a key regulator of the expression level of RANK and RANK ligand (Hanada et al., 2010), inducing an increase in the expression of both molecules. Our results showed effects of disease and treatment in RANK and RANK ligand mRNA expression, increasing their levels in ME7 and TNF-α-treated animals; theoretically supporting the idea of this axis as a key thermoregulator in the current paradigm. However, intracerebroventricular injection of RANKL is reported to produce an acute febrile response via COX-2 activity and PGE2-EP3 activation in the preoptic area of the hypothalamus (Hanada et al., 2009). Therefore whether its increased expression could contribute to the hypothermia observed here, without obvious differential effects on COX-2 requires further investigation.

It is also important to acknowledge that the mice used in this study were all female. This has been done largely to avoid previously observed fighting in males, but it is possible that the oestrus cycle could have some influence on some of the sickness parameters measured here. Indeed it has been observed that RANKL contributes to basal thermoregulation specifically in females (Hanada et al., 2009).

Although TNF-α (initially named cachectin) has significant effects on muscle cachexia, it is more likely that short-term body weight changes are mediated via decreased motivation to consume food which, in LPS and IL-1-induced sickness behavior, is also largely mediated by prostaglandins. Similarly, although the neuroanatomy and molecular mechanisms of sickness-induced hypoactivity remain poorly understood both indomethacin (Teeling et al., 2007) and the COX-1 inhibitor sc-560 (Griffin et al., 2013) reduced measures of LPS-induced sickness behavior and cognitive deficits without effect on systemic or central TNF-α. Those data suggest that prostaglandins would likely to act downstream of TNF-α but it will be necessary to test whether any or all of the current TNF-induced effects are mediated by prostaglandins.

### 4.3 Impact on underlying neurodegenerative pathology

Systemically applied LPS exacerbates existing pathology and accelerates functional decline in multiple models of neurodegeneration including prion disease, amyotrophic lateral sclerosis, Parkinson’s disease and Alzheimer’s disease (Sheng et al., 2003, Nguyen et al., 2004, Pott-Godoy et al., 2008, Cunningham et al., 2009) and there is evidence that acute illness, infection and inflammatory co-morbidity contribute to cognitive decline in Alzheimer’s disease (Yaffe et al., 2004, Holmes et al., 2009, Pandharipande et al., 2013, Widmann and Heneka, 2014). While many systemic inflammatory molecules may exacerbate cognitive decline, the role of systemic TNF-α is currently of particular interest. Elevated baseline TNF-α is associated with MCI conversion to AD (Tarkowski et al., 2003) and with lower hippocampal volume (Sudheimer et al., 2014) suggesting increased systemic TNF-α may influence hippocampal neurodegeneration. In a clinical population of AD patients, elevated baseline systemic TNF-α, when combined with an observed systemic inflammatory event (SIE), was associated with a 10-fold greater rate of cognitive decline over 6 months (Holmes et al., 2009). These studies have lead to a small trial of the TNF-α-blocking monoclonal antibody etanercept in AD patients, which showed good safety and a trend toward improved cognitive status despite being underpowered to detect such effects (Butchart et al., 2015). However, in the current study systemically elevated TNF-α did not induce measureable *de novo* pathology. It is important to acknowledge a weakness of the current experimental design in that the ME7 animals models used here showed very significant synaptic and neuronal pathology prior to TNF-α challenge. If one assumes a relatively modest *de novo* injury, even if occuring selectively in the already degenerating brain, it would be predicted to be relatively difficult to detect on a background of very significant pathology, with the associated variability between different animals. We observed inter-individual differences in severity of pathology, evident in apparently increased Caspase-3 and IBA1 in ME7 + TNFα at 9 h, these were generally within the range of this inter-individual variability. Nonetheless, we have previously shown that acute exacerbation of neuropathology is detectable after systemic LPS (at 500 μg/kg; Cunningham et al., 2005) and we have repeated this observation here. However blocking systemic TNF-α during LPS exposure was not sufficient to block *de novo* cell death in the hippocampus of these animals. Thus, it appears that acutely elevated TNF-α is not sufficient to induce *de novo* hippocampal cell death and neither is systemic TNF-α essential for hippocampal cell death induced by LPS.

It may be significant that elevated baseline TNF-α was a stronger predictor of decline than acute SIE in AD patients (Holmes et al., 2009) and one might predict that chronic elevation of peripheral TNF-α levels would be a better strategy to fully address the hypothesis that elevated systemic TNF-α is a contributor to chronic neurodegeneration and cognitive decline (as opposed to acute cognitive dysfunction occuring during acute inflammatory episodes). Systemic TNF-α has also been shown to mediate the sickness behavior, microglial activation and monocyte infiltration observed during bile duct ligation (liver injury)-induced systemic inflammation (D’Mello et al., 2009). The liver injury induced by bile-duct ligation may constitute a useful, clinically relevant, model scenario with which to produce a more sustained systemic TNF-α response that would alter microglial activation and indeed monocyte infiltration to the brain and may produce measurable impacts on the evolution of neuropathology in the degenerating brain.

## 5 Conclusion

The data indicate that acutely elevated TNF-α has robust acute effects on brain function in the degenerating brain, which may be highly relevant for illness-induced exacerbations of brain dysfunction including depression, delirium and post-operative cognitive dysfunction but levels of this pro-inflammatory cytokine may need to be more sustained to significantly impact on underlying neurodegeneration.

## Figures and Tables

**Table 1:** Primer pair/probe sequences. Where no probe sequence is shown, SYBR green dye was used instead.

Gene	Access. num.	Length	Sequence
COX-1	NM_008969	70 bp	Forward: 5′-CCAGAACCAGGGTGTCTGTGT-3′
			Reverse: 5′-GTAGCCCTGCGAGTACAATC-3′
			Probe: 5′-CGCTTTGGCCTCGACAACTACCAGTG-3′
COX-2	NM_011198.4	128 bp	Forward: 5′-TGGGTGTGAAGGGAAATAAGGA-3′
			Reverse: 5′-GAAGTGCTGGGCAAAGAATG-3′
CCL2	NM_011333	81 bp	Forward: 5′-GTTGGCTCAGCCAGATGCA-3′
			Reverse: 5′-AGCCTACTCATTGGGATCATCTTG-3′
			Probe: 5′-TTAACGCCCCACTCACCTGCTGCTACT-3′
IL-1β	M15131	69 bp	Forward: 5′-GCACACCCACCCTGCA-3′
			Reverse: 5′-ACCGCTTTTCCATCTTCTTCTT-3′
			Probe: 5′-TGGAGAGTCTGGATCCCAAGCAATACCC-3′
RANK	NM_009399.3	63 bp	Forward: 5′-TCGTCCACAGACAAATGCAAA-3′
			Reverse: 5′-GTGTGCTTCTAGTTTCCAAGGA-3′
			Probe: 5′-CTTGGACCAACTGCA-3′
RANK Ligand	NM_011613.3	112 bp	Forward: 5′-GGGGGCCGTGCAGAAGGAAC-3′
			Reverse: 5′-CTCAGGCTTGCCTCGCTGGG-3′
TNF-α	M11731	149 bp	Forward: 5′-CTCCAGGCGGTGCCTATG-3′
			Reverse: 5′-GGGCCATAGAACTGATGAGAGG-3′
			Probe: 5′-TCAGCCTCTTCTCATTCCTGCTTGTGG-3′
GAPDH	NM_008084.2	65 bp	Forward: 5′-GACGGCCGCATCTTCTTGT-3′
			Reverse: 5′-CACACCGACCTTCACCATTTT-3′
			Probe: 5′-CAGTGCCAGCCTCGTCCCGTAGA-3′
